# Polio Field Census and Vaccination of Underserved Populations — Northern Nigeria, 2012–2013

**Published:** 2013-08-23

**Authors:** Saheed O. Gidado, Patrick M. Nguku, Chima J. Ohuabunwo, Ndadilnasiya E. Waziri, Andrew Etsano, Mustapha Z. Mahmud, Faisal M. Shuaib, Charles K. Korir, Pascal Mkanda, Peter B. Bloland, Lisa E. Esapa, Brian C. Kaplan, Frank J. Mahoney, Eric E. Mast, Adamma C. N. Mba-Jonas, Ikechukwu U. Ogbuanu, Alicia G. Ruiz, Steve G. Wassilak, Eric S. Wiesen

**Affiliations:** African Field Epidemiology Network; Nigerian National Primary Health Care Development Agency; Federal Ministry of Health, Nigeria; World Health Organization Country Office, Nigeria; Global Immunization Div, Center for Global Health, CDC

In 2012, the World Health Assembly declared completion of polio eradication a public health emergency (1,2). However, wild poliovirus (WPV) transmission remains endemic in three countries (Afghanistan, Nigeria, and Pakistan) (2–4). In Nigeria, the National Stop Transmission of Polio (N-STOP) program, under the umbrella of the Nigerian Field Epidemiology and Laboratory Training Program (FELTP), has been developed to implement innovative strategies that address the remaining polio eradication challenges in Nigeria. One N-STOP initiative focuses on locating and vaccinating children aged <5 years in remote nomadic, scattered, and border populations in northern Nigeria, where low polio vaccination coverage likely contributes to ongoing WPV transmission. During August 2012–April 2013, N-STOP conducted field outreach activities that enumerated 40,212 remote settlements, including 4,613 (11.5%) settlements never visited by vaccination teams during previous polio supplemental immunization activities (SIAs). Enumeration resulted in documentation of 906,201 children aged <5 years residing in these settlements, including 53,738 (5.9%) who had never received polio vaccination, and in detection of 211 unreported cases of acute flaccid paralysis (AFP) with onset of illness in the 6 months before enumeration. Sustaining access to underserved populations in remote settlements in future SIAs will increase overall population immunity and should decrease WPV transmission. By providing a flexible and capable workforce consisting of Nigerian citizens, N-STOP is able to support evaluation and implementation of innovative polio eradication strategies in Nigeria while building local public health capacity with a potential to address other public health problems following the eradication of polio from Nigeria.

## The N-STOP Program

Nigeria’s N-STOP program is modeled after the global Stop Transmission of Polio (STOP) program established in 1998 to support the Global Polio Eradication Initiative (5,6). Similar to the STOP program, but operated entirely by Nigerian citizens, N-STOP provides technical assistance in strengthening AFP surveillance; planning, implementation, and monitoring of SIAs; operational research; outbreak investigations; and strengthening of routine infant immunization services under the direction of the Nigeria Polio Emergency Operations Center (EOC). N-STOP is a collaboration of the Nigerian FELTP, the Nigerian National Primary Health Care Development Agency, CDC, the World Health Organization Country Office in Nigeria, the United Nations Children’s Fund, and the U.S. Agency for International Development. FELTP is a 2-year fellowship in applied epidemiology and public health that deploys approximately 40 citizens per year to field assignments in locations throughout Nigeria. As of June 30, 2013, a total of 251 N-STOP officers and 1,782 ad hoc field workers had been trained to support polio eradication and routine immunization activities in local government areas in 17 states identified by the Nigeria EOC as having the highest risk for WPV transmission.

## Outreach to Underserved Communities

Administering polio vaccine to every child is the central aim of the Global Polio Eradication Initiative. However, in a study conducted in Borno State in 2005 among Fulani (the dominant nomadic tribe in northern Nigeria) settlements, 99% of children surveyed had not received polio vaccine or other vaccines in the routine childhood vaccination schedule (WHO State Office, Borno State, Nigeria, unpublished data, 2005). One of the major barriers to reaching the Fulani and other underserved communities in northern Nigeria with polio vaccine and other health services is logistic. These communities reside in sparsely populated parts of Nigeria with no road access. In addition, the nomadic herders follow a seasonal pattern of movement with their cattle, with the result that many of the settlements are temporary. Such movements also increase the possibility that WPV will be spread. WPV transmission among Fulani nomads from polio-affected areas to previously polio-free settlements was documented in Niger State during an outbreak investigation in April 2012, and sporadic WPV cases were reported in local government areas along traditional nomadic routes in northern Nigeria (WHO Country Office, Nigeria, unpublished data, 2012). Moreover, children residing in remote nomadic, scattered, and border settlements lack access to routine infant immunization services (Nigerian National Primary Health Care Development Agency, CDC, NFELTP, unpublished data, 2013). These findings indicate that inclusion of these underserved communities in SIA plans and reaching these communities in each SIA are critical to interrupting WPV transmission in Nigeria.

In July 2012, N-STOP piloted a strategy to improve access to underserved communities using a combination of social engagement meetings with community leaders and a field census conducted before implementation of SIAs. N-STOP officers worked closely with local polio immunization staff members to incorporate all newly discovered settlements into plans for upcoming SIAs. In collaboration with national authorities and WHO, this strategy was scaled up during and after SIAs conducted in August 2012. During the meetings with community leaders, settlement lists used for polio SIA planning by the local health office are compared and harmonized with lists of settlements provided by community leaders. Focal points and guides from within the community are recruited to facilitate community acceptance and access to underserved communities. The field census exercise includes using smart phones enabled with global positioning systems to record the settlements included in the harmonized settlement list, conducting a census of children aged <5 years, actively searching for additional settlements, administering polio vaccine, and searching for children with onset of AFP in the 6 months before the field census.

At the end of the field census, a final list of settlements is shared with local and state public health officials so that newly detected settlements can be incorporated into plans for subsequent SIAs. Relationships with community leaders are maintained to ensure community engagement in future vaccination activities. This phase of the field outreach involved identifying chronically missed settlements, administering OPV when feasible, finding missed AFP cases, and strengthening community engagement with the health system on a sustainable basis. Additional phases of the project will include an evaluation of the consistency of future campaigns reaching these settlements and ensuring the inclusion of these underserved settlements in future SIAs.

During August 2012–April 2013, N-STOP supported a field census of underserved populations in 209 local government areas across 17 states with high risk for polio in northern and central Nigeria. During the field census, 40,212 settlements were enumerated, including 4,631 (11.5%) settlements that had never been visited during an SIA (Table) and 7,637 (19.0%) that had not been visited during the preceding SIA. Among the 906,201 children aged <5 years residing in the underserved settlements enumerated, 89,609 (9.9%) resided in settlements that had never been visited during an SIA. Overall, 53,738 (5.9%) of the 906,201 children enumerated had never received polio vaccine (Table). A total of 211 undocumented AFP cases were identified.

### Editorial Note

The findings described in this report demonstrate that many remote nomadic, scattered, and border settlements have been missed by polio vaccination teams during SIAs in northern Nigeria, including a substantial number of settlements that have never been accessed. These settlements are home to thousands of children aged <5 years who are susceptible to WPV infection. By bringing these settlements to the attention of local health officials, the children are often vaccinated during the field census and also can be included in the local plans for future SIAs. Although outreach to these settlements is often logistically difficult because of terrain, security, and distance from centrally located health facilities, the successful penetration by N-STOP teams shows that remote settlements can be reached. The 211 undocumented AFP cases detected during the field census exercises further validate the importance of this outreach strategy. Planning is under way to enhance AFP surveillance in underserved communities. The community engagement strategy and operational procedures used during this field census have been incorporated into the national polio eradication guidelines for the vaccination of children residing in hard-to-reach settlements, which is a key priority of the 2013 National Polio Eradication Emergency Plan for Nigeria.

In addition to outreach to underserved communities, N-STOP provides operational research capabilities to address evolving polio eradication challenges. For example, N-STOP officers recently supported studies to evaluate 1) why some families refuse polio vaccination, 2) why some children are missed during SIAs, and 3) whether a new polio vaccination team-training package is effective and has gained acceptance. N-STOP officers also have played important roles in enhancing routine infant immunization services by serving as focal points dedicated to improving immunization capacity and in investigations of outbreaks of polio and measles by focusing on assessments of underserved communities.

The findings in this report are subject to at least two limitations. First, comprehensive assessment of the field census was limited by the difficulty of estimating the total population of children aged <5 years within the settlements enumerated, which further prevented a complete count of those children who have never been administered polio vaccine. Thus, the proportion of children aged <5 years with no history of polio vaccination relative to the total number of vaccine-eligible children within each settlement cannot be calculated with certainty. Second, the scattered nature of these settlements prevents conclusive completeness of the field census. Even with active searches for settlements by N-STOP teams that included enumerators and local guides, isolated settlements might have been missed. Use of satellite imagery to attempt to locate all possible settlements before conducting a field census is being considered.

N-STOP represents a successful partnership between FELTP, international health organizations, and the public health community in Nigeria. Through the program, public health capabilities in Nigeria have been greatly enhanced. N-STOP will continue to respond to emerging issues related to the Nigeria polio eradication program. In addition, N-STOP will continue to provide field work and leadership opportunities for public health professionals. After polio is eradicated, N-STOP can serve as both a model and an important source of public health leadership in Nigeria.

What is already known on this topic?

Polio eradication is a global public health priority, and Nigeria is one of three countries where endemic polio virus transmission has yet to be interrupted. Interruption has been impeded by omission of residents within nomadic, scattered, border, and otherwise hard-to-reach settlements from vaccination campaigns. Undervaccination of children in these settlements might be creating a reservoir of susceptible persons in a country where wild poliovirus transmission is ongoing.

What is added by this report?

During August 2012–April 2013, the Nigerian National Stop Transmission of Polio (N-STOP) program conducted field exercises to locate and characterize hard-to-reach and nomadic settlements that might be chronically omitted from polio vaccination campaigns and harbor unvaccinated or undervaccinated children aged <5 years. Through these field exercises, 40,212 remote settlements, including 4,613 (11.5%) never before visited by polio vaccination teams, were enumerated. Of the 906,201 children aged <5 years who resided in these settlements, 53,738 (5.9%) had never received polio vaccine. The outreach effort also resulted in the detection of 211 unreported cases of acute flaccid paralysis with onset of illness in the preceding 6 months.

What are the implications for public health practice?

By facilitating the vaccination of previously unimmunized children in hard-to-reach settlements, NSTOP is helping to close the polio immunity gap in northern Nigeria. Closing the immunity gap in chronically missed populations is vital to polio eradication in Nigeria. The field exercise conducted by N-STOP provides a standardized strategy to find and vaccinate children who have been repeatedly missed by previous campaigns. N-STOP officers can rapidly and effectively implement censuses and other programs, and N-STOP represents a substantial enhancement of public health capabilities in Nigeria.

## Figures and Tables

**TABLE:** Number of settlements and children aged <5 years enumerated and number of children identified with no previous polio vaccination, by census period— northern Nigeria, August 2012–April 2013

	Settlements			Children aged <5 yrs				
		
Census period	No. enumerated	No. not visited previously	(%)	Estimated total population in enumerated LGAs	No. enumerated	(%*)	No. identified with no polio vaccination	(%†)
August 2012	10,329	1,578	(15.3)	1,803,199	223,663	(12.4)	7,839	(3.5)
Oct–Nov 2012	9,575	848	(8.9)	1,222,161	205,100	(16.8)	14,123	(6.9)
December 2012	5,145	232	(4.5)	963,305	173,166	(18)	9,360	(5.4)
February 2013	5,072	662	(13.1)	1,403,260	103,573	(7.4)	6,304	(6.1)
March 2013	5,833	844	(14.5)	1,376,508	101,633	(7.3)	9,615	(9.5)
April 2013	4,258	467	(11.0)	1,295,475	99,066	(7.6)	6,497	(6.6)
**Total**	**40,212**	**4,631**	**(11.5)**	**8,063,878**	**906,201**	**(11.0)**	**53,738**	**(5.9)**

**Abbreviation:** LGAs = local government areas.

* Percentage of estimated total population of children aged <5 years in enumerated LGAs.

† Percentage of the number of children aged <5 years enumerated.
